# Rare Posterior Mediastinal Müllerian Cyst Resected by VATS: Case Report and Literature Review

**DOI:** 10.3390/jcm15072773

**Published:** 2026-04-07

**Authors:** Małgorzata Edyta Wojtyś, Wiktoria Skórka, Miłosz Podsiadło, Konrad Ptaszyński, Katarzyna Rodak, Dominik Jakubowski, Tomasz Grodzki

**Affiliations:** 1 Department of Thoracic Surgery and Transplantation, Pomeranian Medical University in Szczecin, 70-891 Szczecin, Poland; 2 Student Scientific Club of Department of Thoracic Surgery and Transplantation, Pomeranian Medical University in Szczecin, 70-204 Szczecin, Poland; 3 Department of Pathology, Regional Hospital SPWSZ in Szczecin, 71-455 Szczecin, Poland; 4 Department of Diagnostic Imaging and Interventional Radiology, Pomeranian Medical University in Szczecin, 71-252 Szczecin, Poland

**Keywords:** Müllerian cyst, VATS, Hattori cyst, mediastinal cyst, case report

## Abstract

**Background**: Müllerian cysts of the posterior mediastinum are exceedingly rare benign lesions that closely resemble other mediastinal cysts on imaging, making preoperative diagnosis difficult. **Methods**: Here, we report the case of a 36-year-old woman in whom a paravertebral cystic lesion at the T8 level was incidentally detected during evaluation of nonspecific pain in the right upper limb. Laboratory tests and chest computed tomography were unremarkable apart from the well-circumscribed homogeneous mediastinal cyst. The lesion was completely excised via video-assisted thoracoscopic resection. **Results**: Histopathological and immunohistochemical evaluation confirmed Müllerian differentiation. The postoperative course was uneventful, and no recurrence was observed during 10 months of follow-up. **Conclusions**: This case highlights the importance of considering Müllerian cysts in the differential diagnosis of posterior mediastinal lesions and demonstrates that complete resection is both diagnostic and curative. A review of the currently available literature offers up-to-date diagnostic and therapeutic approaches for these exceptionally rare lesions.

## 1. Introduction

The mediastinum is an anatomical compartment of the thoracic cavity in which both neoplastic and non-neoplastic lesions with diverse histopathological characteristics may develop [1]. Cystic lesions constitute a significant proportion of mediastinal lesions, and their differential diagnosis includes bronchogenic, esophageal, pericardial, and neurogenic cysts [2,3]. Most of these lesions are detected incidentally during imaging performed for unrelated reasons, and their clinical presentation largely depends on the location and size of the lesion [4]. In recent decades, extremely rare cysts arising from residual paramesonephric tissue, referred to as Müllerian or Hattori cysts, have been increasingly reported [5].

Müllerian cysts represent a relatively recent entity in clinical and diagnostic practice. They were first described in 2005 by Hattori, who identified a characteristic immunohistochemical profile associated with the expression of estrogen receptors (ERs) and progesterone receptors (PRs) [6]. Since then, several dozen case reports have been published worldwide, contributing to a better understanding of the clinical and histopathological features of these cysts [5]. However, diagnosis of such cysts is exceptionally rare and occurs almost exclusively in middle-aged women in the perimenopausal period [5,7].

Nearly all reported cases of Müllerian cysts occur in the posterior mediastinum, adjacent to the spine and within the paravertebral space [5]. The etiopathogenesis of these lesions has not been fully elucidated. The most widely accepted hypothesis is ectopic development of residual Müllerian ducts within the mediastinum that undergo cystic transformation under hormonal influences [6]. The presence of hormone receptors in the epithelial lining of the cyst is considered the most important feature distinguishing Müllerian cysts from other mediastinal cysts [6,7].

Preoperative diagnosis of Müllerian cysts remains challenging. Their radiological appearance on computed tomography (CT) and magnetic resonance imaging (MRI) does not significantly differ from that of other posterior mediastinal cysts, making preoperative identification virtually impossible [8]. These lesions are typically well-circumscribed and homogeneous, without features suggestive of invasion into adjacent structures. The cysts are often small and discovered incidentally, such as during evaluation of chest pain or trauma-related imaging [9]. Laboratory tests are usually unremarkable, and physical examination rarely reveals any significant abnormalities [10].

Definitive diagnosis is possible only by histopathological evaluation using an extended immunohistochemical panel after surgical excision of the lesion. Percutaneous biopsy often provides limited diagnostic material, which prevents comprehensive immunohistochemical analysis and hinders full assessment of the cyst wall structure. This procedure also carries the risk of complications, such as cyst rupture, infection, or bleeding. The typical immunophenotype of Müllerian cysts includes ciliated columnar epithelium positive for ERs, PRs, *paired box gene 8 (PAX8),* and Wilms Tumor 1 (WT1) [11]. Cytokeratin 7 (CK7) expression is also commonly observed, whereas markers characteristic of neurogenic or pulmonary lesions are absent [12]. This immunohistochemical profile is crucial for distinguishing Müllerian cysts from neurogenic, bronchogenic, or esophageal cysts.

The treatment of Müllerian cysts is surgical and consists of complete resection of the lesion, typically via minimally invasive video-assisted thoracoscopic surgery (VATS) [13]. This method allows for simultaneous diagnostic tissue acquisition and a definitive cure. In the case reports published to date, no recurrence or malignant transformation has been reported, and the postoperative course has generally been uneventful. Current evidence suggests that complete excision is curative, and the long-term prognosis is excellent.

Despite the growing number of reports, Müllerian cysts remain an exceptionally rare diagnosis, and most available data are based on isolated case reports [14]. Consequently, their relevance in routine clinical practice remains limited, and awareness of this entity is largely confined to thoracic surgeons, radiologists, and pathologists. Given the nonspecific clinical and radiological presentation, recognition of the characteristic histopathological and immunohistochemical features is essential for establishing an accurate diagnosis.

Here, we present a case with a posterior mediastinal Müllerian cyst and review the current literature regarding the diagnostic and therapeutic approaches to this extremely rare clinical entity.

## 2. Case Presentation

A 36-year-old woman with a BMI of 25.21 kg/m^2^, occasional alcohol use but no tobacco use, and who was employed in an office setting presented to the emergency department with right upper limb pain.

The patient had no history of chronic illnesses or prior surgical procedures. She was not taking any long-term medications. Family history revealed gastric cancer in the patient’s father and grandmother. Physical examination on admission revealed no significant abnormalities. Laboratory tests performed at admission, including hematological, biochemical, and coagulation parameters, were within normal limits.

**Figure 1:**
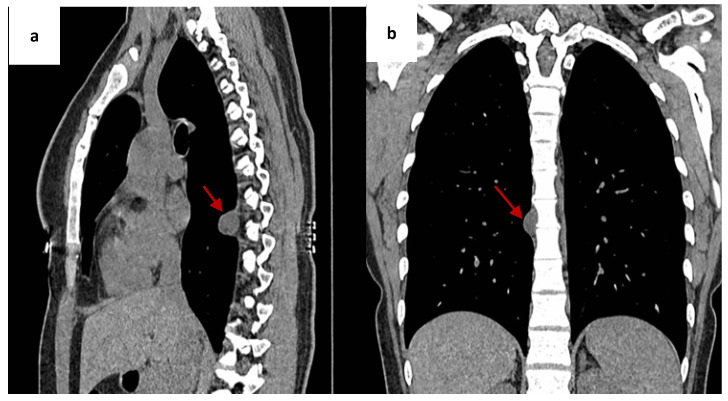
(**a**) Chest CT (sagittal plane) showing a paravertebral Müllerian cyst (arrow) in the right posterior mediastinum. (**b**) Chest CT (coronal plane) showing the Müllerian cyst (arrow).

A right-sided paravertebral lesion at the T8 level was detected incidentally during imaging for pain in the right upper limb. Chest radiography demonstrated a well-circumscribed lesion in the posterior mediastinum. Chest CT demonstrated a paravertebral cystic lesion measuring 9 × 20 × 19 mm at the T8 level with homogeneous fluid density and no signs of invasion into adjacent structures (Figure 1).

Abdominal ultrasound showed no lymphadenopathy or organ abnormalities in the liver, kidneys, pancreas, spleen, or gallbladder. Spirometry with a bronchodilator test did not reveal any significant ventilatory impairment.

Given the characteristics and location of the lesion, the patient qualified for surgical treatment. The patient was admitted to the Department of Thoracic Surgery for surgical management of the lesion. Resection of the posterior mediastinal cyst was performed via right-sided VATS in the operating room under full sedation. A smooth-walled cyst measuring 19 × 12 mm was identified intraoperatively and completely excised.

**Figure 2:**
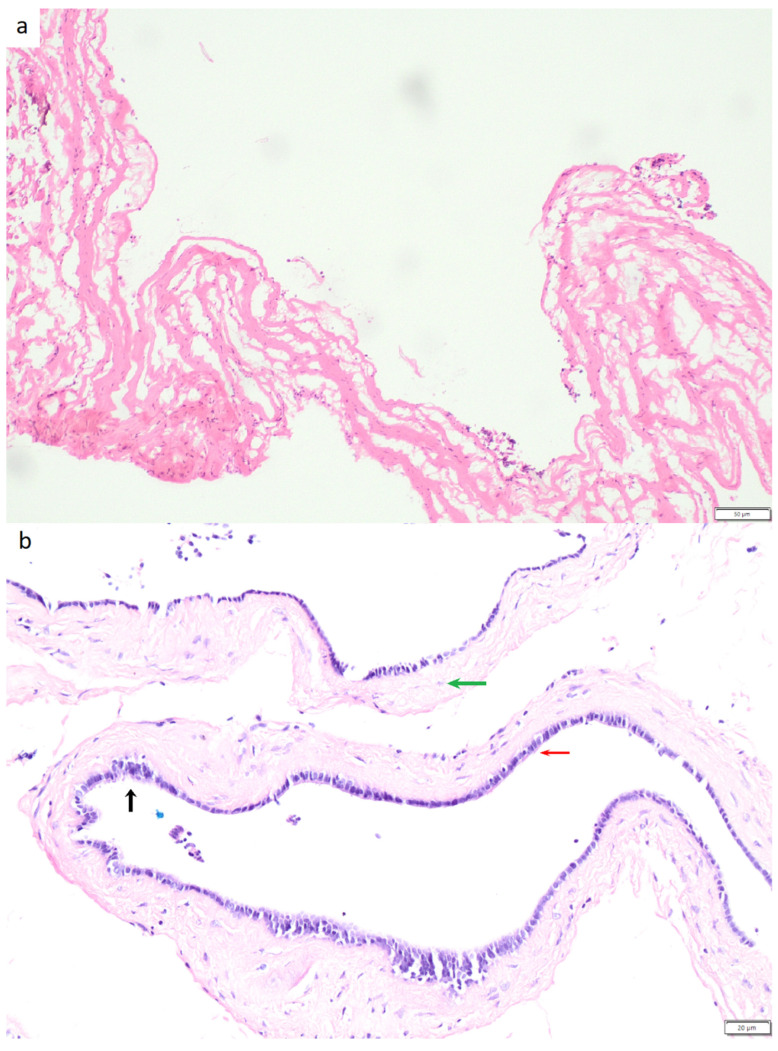
(**a**) Intraoperative examination of the cyst wall (frozen section), 10×, H&E stain. (**b**) Histological architecture of the cyst wall showing single-layer columnar epithelium (red arrow), pseudostratified epithelium (black arrow), and smooth muscle fibers (green arrow), 20×, H&E stain.

The cyst had a 2 mm thick wall and was submitted for intraoperative evaluation. Tissue samples were taken from the cyst wall for histopathological examination, which revealed a simple cyst wall with hyalinizing stroma (Figure 2a).

After the intraoperative assessment, the specimen was fixed in 10% buffered formalin, and additional sections were obtained. Histopathological examination demonstrated a benign cyst lined by single-layer columnar and pseudostratified epithelium (Figure 2b) with smooth muscle fibers and small neural structures visible within the wall.

**Figure 3:**
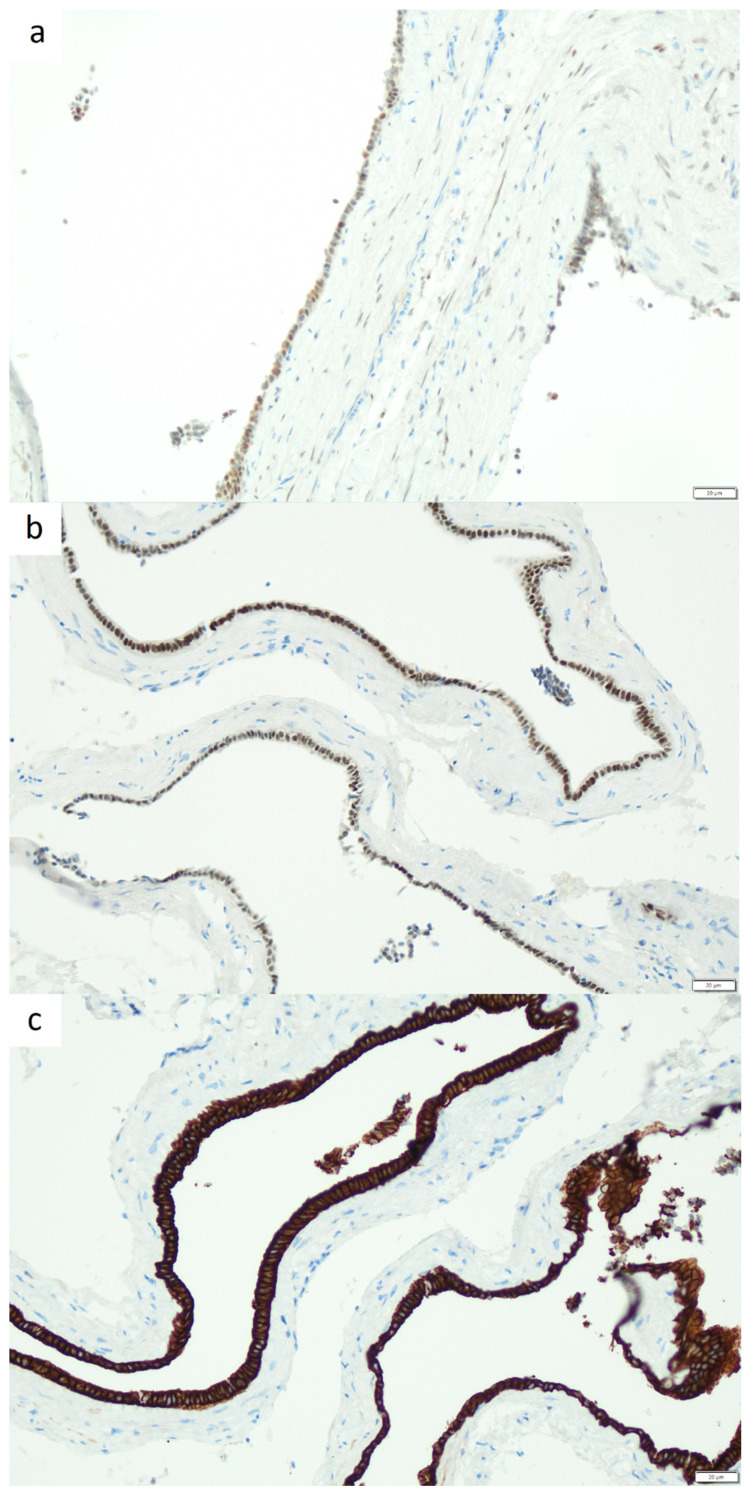
(**a**) Immunohistochemistry for estrogen receptors, 20×. (**b**) Immunohistochemistry for *PAX-8*, 20×. (**c**) Immunohistochemistry for CK7, 20×.

Immunohistochemical analysis demonstrated expression of ER, *PAX8*, CK7, desmin, and SMA. The samples were negative for TTF-1, CK20, and S100 (Figure 3).

The morphology together with the immunophenotype corresponded to a benign mediastinal cyst with Müllerian differentiation.

The postoperative course was uneventful. The pleural drain was removed on the first postoperative day. The patient was discharged on postoperative day 3 in good general condition without respiratory complaints. The 10-month follow-up revealed no evidence of recurrence. The patient’s perspective regarding the treatment they received was positive.

## 3. Discussion

A Müllerian mediastinal cyst (MMC), also known as a Hattori cyst, is a rare benign lesion located in the posterior mediastinal compartment and represents <1% of mediastinal lesions [15]. Only about 50 cases have been reported in the literature to date [5]. Table 1 summarizes the reported cases of Müllerian cysts available in the current literature, along with the ages of the patients.

MMCs are estimated to account for approximately 5.5–15.8% of all cystic lesions of the mediastinum [6,12,19]. In a small series of 19 mediastinal cysts, 3 were reported to be MMCs (15.8%) [6]. All mediastinal nonneoplastic cysts received in one laboratory in France (163 cysts) were re-reviewed, and 5.5% had Müllerian differentiation [19].

Although the exact pathogenesis of MMCs has not been fully established, the consistent immunohistochemical profile observed across reported cases supports a Müllerian origin. Expression of ER, PR, *PAX8*, and WT1 mirrors that of Müllerian-derived tissues and strongly argues against alternative explanations, such as mesothelial metaplasia. These markers are particularly useful in differentiating MMCs from bronchogenic cysts, which are characteristically negative for ERs and PRs and represent one of the primary differential diagnoses. In addition to an epithelial lining, the cyst wall of a Hattori cyst contains smooth muscle fibers. The absence of cartilage and glandular structures further distinguishes it from bronchogenic cysts [21]. A Hattori cyst is composed of epithelium derived from the paramesonephric (Müllerian) ducts, from which the female reproductive organs develop during embryogenesis. It is not entirely clear why Müllerian tissue may be present in the mediastinum, but it is suspected to be due to abnormal distribution of Müllerian tissue during development [21].

Awareness of Müllerian cysts is essential because their appearance on imaging could be challenging to distinguish from other posterior mediastinal lesions, including neurogenic tumors and bronchogenic cysts, and failure to include MMC in the differential may delay definitive diagnosis. Furthermore, progressive enlargement carries risks of spinal cord or nerve root compression and intracystic hemorrhage. The inability to exclude malignant transformation on imaging provides additional justification for surgical resection even in asymptomatic patients.

Despite the increasing number of reported cases, most of these tumors are detected incidentally due to their paucisymptomatic nature. However, a Müllerian cyst may induce certain symptoms, most commonly related to its location, such as back pain, cough, or dysphagia [5,15,19]. In the present case, the lesion was identified during evaluation for nonspecific pain in the right upper limb, which is consistent with previously reported patterns. The nonspecific nature of these symptoms, combined with their overall rarity, prevents the establishment of a consistent or well-defined symptomatological pattern.

Imaging studies play a crucial role in the detection, differential diagnosis, and assessment of mediastinal lesions. A key initial step in evaluating mediastinal pathology is determining in which mediastinal compartment the lesion is located, as this significantly narrows the differential diagnosis. For this purpose, optimal assessment involves obtaining chest X-rays in two projections: posteroanterior and lateral. A characteristic radiographic feature that helps establish the relationship between a mediastinal mass and the pulmonary hilum is the hilum overlay sign. In addition, an MMC is characterized by several radiological features that may suggest the correct diagnosis. These lesions are typically located in the posterior mediastinum, most often in the paravertebral region at T3–T6. In the majority of cases, MMCs present as unilocular, thin-walled, well-circumscribed cystic lesions. They also frequently exhibit a somewhat flattened contour, which may help distinguish them from bronchogenic cysts. Chest radiography also plays a role in the postoperative setting, where it is used to assess for potential complications [22].

Another imaging modality used in the evaluation of mediastinal masses is CT. This method is often employed as the diagnostic study of choice following initial assessment by chest radiography. The advantage of CT lies in its high spatial resolution, which allows for precise evaluation of the lesion’s location and its anatomical relationships with adjacent structures, making it useful for preoperative planning. CT also enables detailed assessment of the lesion size, attenuation characteristics, the presence of calcifications, macroscopic fat, and the pattern of contrast enhancement. However, CT has a lower capability for soft-tissue characterization compared with MRI and is associated with exposure to ionizing radiation. In the case of cystic lesions, CT typically demonstrates low radiological density, usually ranging from 0 to 20 Hounsfield units (HU) [23]. In some cases, increased attenuation (20 to 100 HU) may be observed, which makes it more difficult to differentiate complicated cysts from solid lesions [24]. This phenomenon is associated with proteinaceous, hemorrhagic, or calcified contents within the cyst. Such variability means that, in some cases, the exact nature of the cyst can only be determined using MRI. When intravenous contrast is administered, MMCs typically do not demonstrate contrast enhancement.

MRI allows for the most accurate characterization of mediastinal lesions. Appropriate use of MRI sequences enables differentiation of solid and cystic masses, as well as the identification of cystic or necrotic components within solid lesions. In the evaluation of mediastinal pathology, MRI is often considered a diagnostic problem-solving tool, particularly when CT findings are inconclusive. The typical appearance of MMCs on MRI is that of a well-circumscribed lesion demonstrating fluid signal intensity, which comprises a low signal on T1-weighted images and a high signal on T2-weighted images. However, in rare cases, variable T1 signal intensity may be observed due to proteinaceous or hemorrhagic contents within the cyst [25]. Typically, these lesions do not demonstrate contrast enhancement after the administration of gadolinium-based agents. Therefore, when a relatively homogeneous cystic lesion is identified in the posterior mediastinum with imaging characteristics suggesting fluid content, the possibility of an MMC should be considered.

Although CT and MRI can substantially narrow the differential diagnosis, definitive diagnosis requires histopathological and immunohistochemical confirmation. Surgical resection is considered the treatment of choice and serves both diagnostic and therapeutic purposes. Minimally invasive approaches, particularly VATS, allow for complete excision while minimizing postoperative morbidity. To date, no publications have reported recurrence following complete resection, which supports the concept that MMCs behave as a benign lesion with an excellent prognosis [5,17]. VATS also provides excellent visualization of the mediastinum and allows for the acquisition of adequate tissue samples for immunohistopathological diagnosis [26,27]. Robotic approaches, such as the Da Vinci^R^ (Intuitive Surgical) system, as well as thoracotomy, are used in select cases [19]. In this case report, VATS was the method of choice rather than thoracotomy, which accelerated the patient’s recovery.

The current literature does not report any postoperative complications among patients during follow-up [13,15,16]. In addition, no malignant transformation of MMC has been reported in the literature. However, due to the rarity of this entity and the limited follow-up data available, the long-term biological behavior of these lesions remains to be completely characterized [28].

The CARE guidelines were followed in this paper. The limitation of this case report is the small sample size. However, it is a well-documented rare clinical case with a comprehensive literature review.

## 4. Conclusions

This case report highlights the need to consider Müllerian cysts in the differential diagnosis of posterior mediastinal cystic lesions. Due to their nonspecific clinical and radiological appearance, definitive diagnosis relies on histopathological and immunohistochemical evaluation. Complete VATS excision remains both diagnostic and curative.

## Figures and Tables

**Table 1:** Comparison of reported cases of posterior mediastinal Müllerian cysts.

Treatment/Follow-Up	Markers	Symptoms	Location of Cyst	Age (Years)/Sex/BMI (kg/m^2^)	Author/Year
VATS/12 months, doing well	ER + *PAX8 +* PR+	None	Left posterior mediastinum	40/F/no data	Atsushi Sekimura/2018 [9]
Robotic resection/3 weeks, stable, no complaints	ER + *PAX8+*	Back pain	Right posterior mediastinum	60/F/no data	Christopher Sullivan/2022 [15]
VATS/no data	ER + PR + CK7 + *PAX8+*	None	In front of T10	41/F/17	Motoaki Yasukawa/2018 [16]
VATS/12 months, no recurrence	ER + PR + WT1+	None	Left paravertebral, T10	22/F/no exact data (obesity)	Jung Eun Lee/2018 [13]
VATS/12 months, no recurrence	ER + *PAX8 +* WT1 + PRC+	Low back pain	Left posterior mediastinum	47/F/21.8	Aliye Abulizi/2024 [10]
VATS/12 months, no recurrence	ER + PR + WT1 + *PAX8+*	Pharyngeal discomfort when coughing up sputum	Left posterior mediastinum	54/F/22.3	Aliye Abulizi/2024 [10]
VATS/6 months, no recurrence	*PAX8 +* WT1 + ER+	None	Right posterior mediastinum	42/F/no exact data (not overweight)	Kai Kang/2022 [17]
Robotic resection/no data	ER + PR + *PAX8 +* WT1 + PR+	Shortness of breath	Posterior mediastinum	52/F/no data	Matthew Simmon/2013 [5]
No data/no data	ER + WT1+	No data	Left posterior mediastinum	47/F/no data	Matthew Simmon/2013 [5]
VATS/no data	ER+	None	Posterior mediastinum	53/F/no data	Satoru Kobayashi/2012 [18]
Thoracotomy/no data	ER + PR+	Chest pain, dysphagia	Left posterior mediastinum	40/F/no data	Thomas-de-Montepreville/2007 [19]
Thoracotomy/no data	ER + PR+	Cough	Left posterior mediastinum	46/F/no data	Thomas-de-Montepreville/2007 [19]
Thoracotomy/no data	ER + PR+	Cough	Right posterior mediastinum	47/F/no data	Thomas-de-Montepreville/2007 [19]
Thoracotomy/no data	ER + PR+	None	Left posterior mediastinum	48/F/no data	Thomas-de-Montepreville/2007 [19]
Thoracotomy/ no data	ER + PR+	None	Right posterior mediastinum	50/F/no data	Thomas-de-Montepreville/2007 [19]
Thoracotomy/no data	ER + PR+	None	Left posterior mediastinum	51/F/no data	Thomas-de-Montepreville/2007 [19]
Thoracotomy/no data	PR+	None	Left posterior mediastinum	56/F/no data	Thomas-de-Montepreville/2007 [19]
Thoracotomy/no data	-	Cough	Paravertebral	58/F/no data	Thomas-de-Montepreville/2007 [19]
Thoracotomy/no data	-	Chest pain	Right posterior mediastinum	59/F/no data	Thomas-de-Montepreville/2007 [19]
VATS/no data	ER + PR + WT1 + CK7+	None	Posterior mediastinum	52/F/33	Evgeny B. Topolnitskiy/2026 [20]
VATS/no data	ER + PR + WT1 + CK7+	Back pain	Left posterior mediastinum	51/F/30	Evgeny B. Topolnitskiy/2026 [20]

Abbreviations: VATS, video-assisted thoracoscopic surgery; ER, estrogen receptor; PR, progesterone receptor; PRC, prolactin receptor; WT1, Wilms tumor 1, *PAX8*, *paired box gene 8.*

## Data Availability

The data presented in this study are available in this article.

## Abbreviations

The following abbreviations are used in this manuscript:

ER	estrogen receptor
*PAX8*	*paired box gene 8*
WT1	Wilms tumor 1
CK7	cytokeratin 7
VATS	video-assisted thoracoscopic surgery
SMA	alpha-smooth muscle actin
TTF-1	thyroid transcription factor-1
CK20	cytokeratin 20
S100	S100 proteins
